# Prevalence of sleep disturbances in children and adolescents during COVID-19 pandemic: a meta-analysis and systematic review of epidemiological surveys

**DOI:** 10.1038/s41398-023-02654-5

**Published:** 2024-01-08

**Authors:** Hong Cai, Pan Chen, Yu Jin, Qinge Zhang, Teris Cheung, Chee H. Ng, Yu-Tao Xiang, Yuan Feng

**Affiliations:** 1 Unit of Medical Psychology and Behavior Medicine, School of public health, Guangxi Medical University, Nanning, Guangxi China; 2 Unit of Psychiatry, Department of Public Health and Medicinal Administration, & Institute of Translational Medicine, Faculty of Health Sciences, University of Macau, Macao SAR, China; 3 Centre for Cognitive and Brain Sciences, University of Macau, Macao SAR, China; 4 College of Education for the Future, Beijing Normal University, Beijing, China; 5 Beijing Key Laboratory of Mental Disorders, National Clinical Research Center for Mental Disorders & National Center for Mental Disorders, Beijing Anding Hospital, Capital Medical University, Beijing, China; 6 School of Nursing, Hong Kong Polytechnic University, Hong Kong SAR, China; 7 Department of Psychiatry, The Melbourne Clinic and St Vincent’s Hospital, University of Melbourne, Richmond, VIC Australia

**Keywords:** Psychiatric disorders, Scientific community

## Abstract

The COVID-19 pandemic and the ensuing widespread lockdown measures have had a negative impact on the mental health of children and adolescents. We thus conducted a meta-analysis of the worldwide prevalence of sleep disturbances in children and adolescents during the COVID-19 pandemic. We performed a systematic literature search of the major international (PubMed, PsycINFO, Web of Science) and Chinese (Chinese Nation Knowledge Infrastructure (CNKI) and WANFANG) databases from their commencement dates to 27 December 2022. Altogether, 57 articles covering 206,601 participants were included in the meta-analysis. The overall prevalence of sleep disturbances was 34.0% (95% confidence interval (CI): 28–41%). The prevalence of parent-reported sleep disturbances during the COVID-19 pandemic was significantly higher than that of self-reported (*p* = 0.005) sleep disturbances. Epidemiological studies jointly conducted across Asia and Europe had a higher prevalence of sleep disturbances compared to those conducted in Asia, Europe, America, Oceania, or South America alone (*p* < 0.001). Children had a significantly higher prevalence of sleep disturbances compared to adolescents alone or a mixed cohort of children and adolescents (*p* = 0.022). Meta-regression analyses revealed that mean age (*p* < 0.001), quality evaluation score (*p* < 0.001), and percentage of men (*p* < 0.001) showed negative associations, while time of survey (*B* = 1.82, *z* = 34.02, *p* < 0.001) showed a positive association with the prevalence of sleep disturbances. Sleep disturbances were common in children and adolescents during the COVID-19 pandemic.

## Introduction

Coronavirus disease 2019 (COVID-19) which is caused by severe acute respiratory syndrome coronavirus 2 (SARS-CoV-2), has been declared a pandemic since March 11, 2020 by the World Health Organization (WHO) [1]. As of September 23, 2020, there have been over 230 million COVID-19 cases and more than 4 million deaths caused by COVID-19 [2]. The crucial COVID-19 pandemic preventive measures such as mass lockdowns, social distancing, mask-wearing, frequent hand hygiene, and restriction of school and recreational activities have had a negative influence on the mental health of nearly all populations especially children and adolescents, such as an increased risk of depression, anxiety, post-traumatic stress disorder (PTSD), and sleep problems [3–8].

To reduce the negative impact of adverse mental health and allocate appropriate health resources, understanding the epidemiology of mental health problems and their associated factors is important. In the past years, numerous studies on the mental health impacts on children and adolescents, particularly the occurrence of depressive and anxiety symptoms, have been conducted, with mixed findings [9, 10]. A recent meta-analysis revealed that the pooled prevalence estimates of depressive and anxiety symptoms were 25.2% (95% confidence interval (CI): 21.2–29.7%) and 20.5% (95% CI: 17.2–24.4%), respectively in children and adolescents during the COVID-19 pandemic [11]. Sleep disturbances might be associated with increased stress levels, excessive online activities, reduced peer interactions, increased daytime sleep, and disrupted daily routine and sleep/wake schedule, all of which could increase the risk of loneliness, negative affect, lethargy, and napping behaviors [12].

Although many studies focused on sleep disturbances in children and adolescents, the findings varied greatly between studies with very few meta-analyses published. One meta-analysis reported that the prevalence of sleep problems was 35.7% (95% CI: 29.4–42.4%) in the general population during the COVID-19 pandemic [13]. In a recent meta-analysis of major sub-populations [14], the prevalence of sleep disturbances was 45.96% [36.90- 55.30%] (*N* = 10) among children and adolescents during the COVID-19 pandemic. Another meta-analysis found that the pooled prevalence of sleep disorders alone was 44% (95% CI: 21%- 68%) in children and adolescents during the pandemic [8], but when more broadly defined, those with sleep disturbances were not included. In another meta-analysis examining sleep disturbances in children and adolescents with and without neurobehavioral disorders, the pooled prevalence of any sleep disturbance was 54% during the COVID-19 pandemic (95% CI: 50–57%) [15]. However, the inclusion of neurobehavioral disorders and only a small number of studies (*N* = 3) might have biased the findings. This gave us the impetus to conduct a meta-analysis on the worldwide prevalence and associated factors of sleep disturbances in children and adolescents during the COVID-19 pandemic.

## Material and methods

### Search strategy and selection criteria

This meta-analysis was conducted based on the Preferred Reporting Items for Systematic Review and Meta-Analyses (PRISMA) [16]. This protocol was registered in the International Platform of Registered Systematic Review and Meta-analysis Protocol (INPLASY) (registration number is INPLASY202190098). Two investigators (HC and PC) independently searched the relevant literature in the major international (PubMed, PsycINFO, and Web of Science) and Chinese databases (Chinese Nation Knowledge Infrastructure (CNKI) and WANFANG) from their commencement dates until 27 December 2022 using the following terms: (Sleep Initiation and Maintenance Disorders [MeSH Terms] OR sleep disturbance OR insomnia OR sleep problem OR sleep disorder OR sleep symptom OR sleep*) AND (adolescent [MeSH Terms] OR child OR children OR preschool OR pediatrics OR infants OR toddlers) AND (2019-ncov* OR 2019ncov* OR 2019n-cov* OR coronaviru* OR corona viru* OR covid OR covid-19 OR covid19* OR novel cov* OR ncov* OR covid-2019 OR covid2019 OR SARS-COV2* OR SARS COV-2* OR SARS COV2* OR SARS COV19 OR SARS COV-19 OR SARS-COV-2019 OR SARS COV 2019 OR SARS COV-2019 OR severe acute respiratory syndrome or severe acute respiratory disease) AND (epidemiology OR prevalence OR rate) (Supplementary Table 1).

### Inclusion and exclusion criteria

The inclusion criteria were developed according to the PICOS acronym as follows: Participants: Children and Adolescents; Intervention: not applicable; Control: not applicable; Outcomes: the prevalence of sleep disturbances or data that could generate prevalence of sleep disturbances during the COVID-19 pandemic; and Study design: epidemiological surveys, including cross-sectional surveys and baseline (cross-sectional) data of cohort studies. There were no restrictions on the measures on sleep disturbances used. When more than one paper was published based on the same dataset, only the one with the largest sample was included.

### Study selection and data extraction

**Fig. 1:**
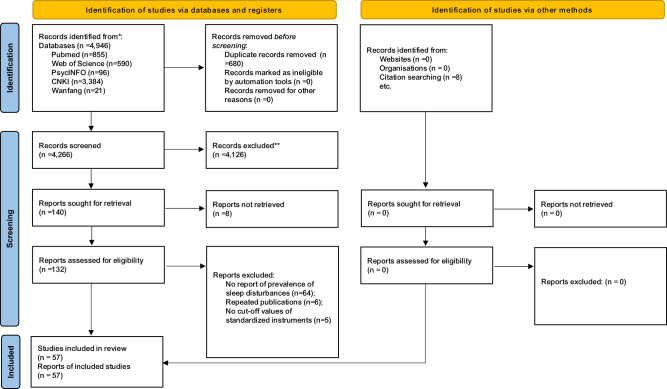
PRISMA flow chart.

The same two investigators independently screened the titles and abstracts, and then read the full texts of relevant papers for eligibility. Moreover, the reference lists of the relevant reviews were checked manually to identify any additional studies. Any uncertainty in the literature search was resolved by a discussion with a third investigator (YTX). The literature search procedure is shown in Fig. 1.

The two investigators independently performed data extraction using a standardized form. Study and participant characteristics such as the first author, year of publication, country, time of the survey, study design, sampling method, mean age, total sample size, sample size of males, and scale used for sleep disturbance were recorded.

### Study quality assessment

Study quality was assessed using an instrument for epidemiological studies [17, 18], with 8 items as follows: (1) target population was defined clearly, (2) probability sampling or entire population surveyed, (3) response rate was equal or >80%, (4) non-responders clearly described, (5) sample representative of the target population, (6) data collection methods standardized, (7) validated criteria used to measure sleep disturbance, and (8) prevalence estimates given with confidence intervals and detailed by subgroups (if applicable). The total score ranged from 0 to 8. Studies with a total score of “7–8” were considered as “high quality”, “4–6” as “moderate quality” and “0–3” as “low quality” [19]. Any uncertainty in the quality assessment was resolved via a discussion with a third investigator (YTX).

### Statistical analysis

This meta-analysis was implemented using the R software [20] and comprehensive meta-analysis (CMA) version 2.0 (Biostat Inc., Englewood, NJ, USA). Due to the different study characteristics between studies, the random-effects model was used to calculate the pooled prevalence of sleep disturbances with their 95% CI. Following previous meta-analyses of epidemiology [21, 22], the raw data were analyzed with the random-effect model. The heterogeneity between studies was assessed with the *I*^2^ statistic, and *I*^2^ > 50% was considered an indication of high heterogeneity [23]. In addition, *τ*^2^ values arising from the random effects model were also used to quantify heterogeneity. The moderating effects of categorical variables (e.g., type of data collection (self-report vs. parent-report), children and/or adolescents, and region classified by the WHO regional classification (Africa/North and South America/Eastern Mediterranean/Europe/South East Asia/Western Pacific) [24]) and continuous variables (e.g., percentage of males, mean age, time of survey and quality evaluation score) on the results were examined using subgroup and meta-regression analyses, respectively. Sensitivity analyses were performed to identify outlying studies by excluding studies one by one. Publication bias was estimated with funnel plots and Begg tests [25]. A *p*-value < 0.05 was considered statistically significant (two-tailed).

## Results

Study and participant characteristics are summarized in Table 1. Out of 4946 articles identified, a total of 57 articles covering 206,601 participants were included in the meta-analysis. The sample size ranged from 28 to 42,077. The mean age of participants ranged from 6.78 to 17.4 years. Study quality assessment scores ranged from 3 to 7; 2 studies were rated as low quality; 51 studies were moderate quality, and 4 were high quality (Supplementary Table 2).

**Table 1:** Characteristics of the studies included in the meta-analysis.

No.	First author (year)	References	Country	Prevalence of sleep disturbance	Time of survey	Study design	Sampling method	Mean age (years)	Total *N*	Males *N*	Scale on sleep disturbance	Study quality total score
1	Liang et al. (2020)	Jing et al. (2020)	China	0.20	2020.02.12–2020.02.20	Cross-sectional	Random sampling	NA	20,579	10,788	Self-made questionnaire	5
2	Wang et al. (2020)	Wang et al. (2020) [5]	China	0.04	2020.03.18–2020.04.18	Cross-sectional	NA	NA	2727	NA	ISI	4
3	Yang et al. (2020)	Yang et al. (2020)	China	0.004	NA	Cross-sectional	Convenience sampling	NA	1889	845	Question	4
4	Bruni et al. (2021)	Bruni et al. (2021)	Italy	0.27	2020.05.07–2020.06.15	Cross-sectional	Convenience sampling	NA	4314	2217	SDSC	4
5	Chi et al. (2021)	Chi et al. (2021)	China	0.38	2020.05.13–2020.05.20	Cross-sectional	Random sampling	15.26	1794	1007	YSIS	7
6	Dondi et al. (2020)	Dondi et al. (2021)	Italy	0.69	2020.09.01–2020.10.15	Cross-sectional	Convenience sampling	NA	6210	NA	SDSC	5
7	Wearick-Silva et al. (2021)	Wearick-Silva et al. (2021)	Brazil	0.51	2020.04.27–2020.07.30	Cross-sectional	Convenience sampling	NA	577	NA	BISQ SDSC PSQI	5
8	Eyuboglu et al. (2021)	Eyuboglu et al. (2021)	Turkey	0.65	2020.07.06–2020.07.10	Cross-sectional	Convenience sampling	8.86	125	51	SDSC	5
9	Fidanci et al. (2021)	Fidanci et al. (2021)	Turkey	0.97	2020.09–2020.10	Cross-sectional	NA	11.32	114	50	SDSC	4
10	Lopez-Gil et al. (2021)	López-Gil et al. (2021)	Brazil	0.49	2020.04.14–2020.04.28	Cross-sectional	Convenience sampling	10.7	495	275	BEARS	5
11	Hu et al. (2021)	Hu et al. (2021)	China	0.20	2020.02.24–2020.02.28	Cross-sectional	Convenience sampling	16.35	2,090	786	CADSS	5
12	Li et al. (2021)	Li et al. (2021)	China	0.21	2020.03.19	Longitudinal study	Convenience sampling	15.87	831	503	ISI	5
13	Liu Y et al. (2021)	Liu et al. (2021a)	China	0.06	2020.06.09–2020.06.28	Cross-sectional	Convenience sampling	13.37	5175	2673	N	6
14	Liu, Z et al. (2021)	Liu et al. (2021b)	China	0.56	2020.02.17–2020.02.19	Cross-sectional	Convenience sampling	NA	1619	NA	CSHQ	6
15	Moulin et al. (2021)	Moulin et al. (2021)	French	0.21	2020.03.24–2020.03.28	Cross-sectional	Convenience sampling	NA	325	NA	Question	4
16	Nakachi et al. (2021)	Nakachi et al. (2021)	Japan	0.40	2020.04.30–2020.05.08	Cross-sectional	Convenience sampling	11.4	535	NA	Question	6
17	Osmanov et al. (2021)	Osmanov et al. (2021)	Russia	0.07	2020.04.02–2020.08.26	Cohort	Convenience sampling	NA	518	348	Question	7
18	Weingart et al. (2021)	Weingart et al. (2021)	USA	0.74	2020.05.07–2020.06.30	Cross-sectional	Convenience sampling	15	590	243	Question	5
19	Resendiz-Aparicio et al. (2021)	Reséndiz-Aparicio (2021)	Mexico	0.20	2020.05.20–2020.05.26	Cross-sectional	Convenience sampling	NA	4000	NA	NA	4
20	Ventura et al. (2021)	Ventura et al. (2021)	Spain	0.16	2020.04.07–2020.04.18	Cross-sectional	Convenience sampling	NA	3464	1727	SDSC	6
21	Wang, L et al. (2021)	Wang et al. (2021)	China	0.31	2020.05.20–2020.06.20	Cross-sectional	Stratified cluster sampling	NA	12,186	6357	Question	7
22	Zhai et al. (2021)	Zhai et al. (2021)	China	0.19	2020.01.09–2020.02.09	Cross-sectional	Convenience sampling	15.94	10,569	5319	PSQI	5
23	Zhou et al. (2020)	Zhou et al. (2020)	China	0.18	2020.03.08–2020.03.15	Cross-sectional	Convenience sampling	NA	9429	4261	PSQI	6
24	Li et al. (2021)	Lin et al. (2021)	China	0.32	2020.02.05–2020.02.23	Cross-sectional	Convenience sampling	NA	74	NA	Question	5
25	Luca et al. (2021)	Pisano et al. (2020)	Italy	0.2	2020.03.21–2020.03.24	Cross-sectional	Convenience sampling	NA	5989	NA	Question	6
26	Kumar et al. (2020)	Saurabh and Ranjan (2020)	India	0.19	NA	Cross-sectional	Convenience sampling	15.4	252	215	Question	4
27	Szwarcwald et al. (2021)	Szwarcwald et al. (2021)	Brazil	0.36	2020.06.27–2020.09.17	Cross-sectional	Convenience sampling	NA	9470	4754	Question	4
28	Ding et al. (2022)	Ding et al. (2022)	China	0.31	2020.06–2020.08	Cross-sectional	Stratified random sampling	NA	307	142	Sleep Disorders Behavior Questionnaire	7
29	Li et al. (2022)	Li et al. (2022)	China	0.84	2022.04.15–2022.05.14	Case-control	Convenience sampling	NA	169	94	CSHQ	4
30	Wang et al. (2022)	Wang et al. (2022)	China	0.09	2021.04–2021.10	Cross-sectional	cluster sampling	NA	5896	3101	CSHQ	6
31	Xu et al. (2022)	Xu et al. (2022)	China	0.42	2020.04–2021.10	Cross-sectional	Convenience sampling	NA	158	69	PSQI	5
32	Zhao et al. (2022)	Zhao et al. (2022)	China	0.71	2020.09–2022.10	Cross-sectional	Random sampling	NA	408	214	CSHQ	6
33	Bacaro et al. (2021)	Bacaro et al. (2021)	Italy	0.77	2020.04–2020.05	Cross-sectional	Convenience sampling	8.1	2361	1148	Question	4
34	Bacaro et al. (2022)	Bacaro et al. (2022)	Italy	0.40	2020.04.14–2020.05.04	Cross-sectional	Convenience sampling	14.95	1146	509	ISI	4
			Italy	0.54	2021.04.12–2021.05.03	Cross-sectional	Convenience sampling	15.68	1406	624	ISI	
35	Becker et al. (2021)	Becker et al. (2021)	United States	0.36	2020.05.16–2020.06.15	Case-control	Convenience sampling	16.28	122	75	SDSC	4
36	Bothe et al. (2022)	Bothe et al. (2022)	Austrian	0.39	2021.02.21–2021.04.19	Cross-sectional	Convenience sampling	NA	2290	818	Question	4
37	Gendler et al. (2022)	Gendler and Blau (2022)	Israel	0.29	2021.01.20–2021.03.20	Cross-sectional	Convenience sampling	13.99	500	235	PROMIS	4
38	Ho et al. (2022)	Ho and Lee (2022)	China	0.30	2021.03	Cross-sectional	Convenience sampling	NA	585	279	Question	4
39	Kaltschik et al. (2022)	Kaltschik et al. (2022)	Austrian	0.27	2022.04.26. 2022.05.24	Cross-sectional	Convenience sampling	16.65	1198	240	ISI	4
40	Lima et al. (2022)	Lima et al. (2022)	Brazil	0.70	2020.08.10–2020.09.30	Cohort study	Convenience sampling	NA	105	48	SDSC	4
41	Ma et al. (2021)	Ma et al. (2021)	China	0.35	2020.02.19–2020.03.05	Cross-sectional	Convenience sampling	12.87	17,740	NA	Question	4
42	Mackenzie et al. (2021)	MacKenzie et al. (2021)	Canada	0.40	2020.06	Cross-sectional	Convenience sampling	8.1	85	44	SDSC	4
43	Mensi et al. (2022)	Mensi et al. (2022)	Italy	0.57	2021.04–2021.07	Cross-sectional	Convenience sampling	15.04	481	184	Question	5
44	Moitra et al. (2022)	Moitra and Madan (2022)	India	0.53	2021.01–2021.07	Cross-sectional	purposive sampling	13.2	1298	692	PSQI	6
45	Monnier et al. (2021)	Monnier et al. (2021)	France	0.37	2020	Cross-sectional	Convenience sampling	NA	5702	2890	Question	4
46	Pieh et al. (2022)	Pieh et al. (2022)	Austrian	0.19	2021.02.03–2021.02.28	Cross-sectional	Convenience sampling	NA	3052	NA	ISI	4
47	Sánchez-Ferrer et al. (2022)	Sánchez-Ferrer et al. (2022)	Spain	0.19	2020.04.22–2020.04.26	Cross-sectional	Convenience sampling	6.78	1501	729	Question	4
48	Scarselli et al. (2022)	Scarselli et al. (2022)	Italy	0.89	2020.04–2020.06	Cross-sectional	Convenience sampling	NA	28	NA	CSHQ	3
49	Sen et al. (2021)	Sen et al. (2021)	Indonesia	0.45	2020.04.28–2020.06.30	Cross-sectional	Convenience sampling	17.4	2932	625	PSQI	4
50	Silver et al. (2022)	Silver et al. (2022)	USA	0.36	2020.11–2021.01	Cross-sectional	Convenience sampling	12.3	33	27	Question	3
51	Ustuner Top and Cam (2022)	Ustuner Top and Cam (2022)	Turkey	0.55	2021.02.01–2021.02.15	Cross-sectional	Convenience sampling	9.16	1040	528	CSHQ	5
52	Van et al. (2022)	van der Velden et al. (2022)	Netherland	0.20	2020.11–2020.12	Cohort	Convenience sampling	NA	251	128	Question	5
53	Xue et al. (2022)	Xue et al. (2022)	China	0.03	2020.04–2020.06	Cross-sectional	cluster sampling	16.45	42,077	19,525	Question	5
54	Zhan et al. (2022)	Zhan et al. (2022)	China	0.12	2020.02.04–2020.02.18	Cross-sectional	Convenience sampling	12.47	1355	714	YSIS	5
55	Zhang et al. (2021)	Zhang et al. (2021)	China	0.17	2020.01–2020.03	Case-control	NA	NA	152	98	SDSC	5
56	Zhou et al. (2021)	Zhou et al. (2021)	China	0.12	2021.04.06–2021.04.14	Cross-sectional	Convenience sampling	16.39	1108	544	SRSS	6
57	Zhu et al. (2022)	Zhu et al.(2022)	China	0.06	2020.06.09–2020.06.29	Cross-sectional	Random	13.37	5175	2673	Question	7

*NA* not available, *SDSC* sleep disturbance scale for children, *YSIS* youth self-rating insomnia scales, *PSQI* Pittsburgh sleep quality index, *CSHQ* children’s sleep habit, *BISQ* brief infant sleep questionnaire, *BEARS* bedtime problems, Excessive daytime sleepiness, Awakenings during the night, Regularity and duration of sleep, and Sleep-disordered breathing sleep screening, *CADSS* Chinese adolescent daytime sleepiness scale, *ISI* insomnia severity index.

### Pooled prevalence of sleep disturbance

**Fig. 2:**
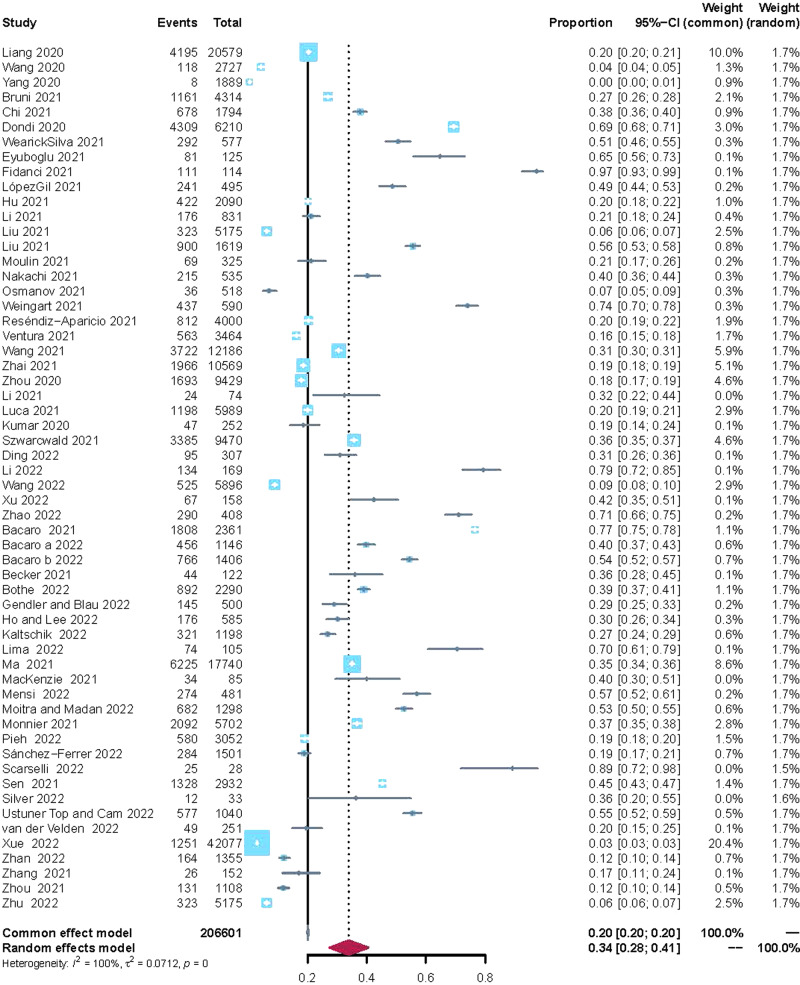
The prevalence of sleep disturbances in children and adolescents during the COVID-19 pandemic.

In the meta-analysis, 57 studies reported the prevalence of sleep disturbances in children and adolescents. The overall prevalence of sleep disturbance was 34.0% (95% CI: 28–41%, *I*^2^ = 100%, *τ*^2^ = 0.071) (Fig. 2).

### Sensitivity analysis and publication bias

Sensitivity analyses did not find any outlying studies that could significantly change the prevalence of sleep disturbances in children and adolescents during the COVID-19 pandemic. The Funnel plot and Begg test did not find significant publication bias with respect to the prevalence of sleep disturbance (Begg test: *z* = 0.21, *p* = 0.83) (Supplementary Fig. 1).

### Subgroup and meta-regression analyses

As shown in Table 2, the type of data collection, region, and inclusion of children and/or adolescents was significantly associated with the prevalence of sleep disturbances. The prevalence of parent-reported sleep disturbances during the COVID-19 pandemic were significantly higher than that of self-reported sleep disturbances (*Q* = 7.948, *p* = 0.005). Epidemiological studies jointly conducted across Asia and Europe had a higher prevalence of sleep disturbances compared to those conducted in Asia, Europe, America, Oceania, and South America alone (*Q* = 23,656, *p* < 0.001). Children had a significantly higher prevalence of sleep disturbances compared to adolescents alone or a mixed cohort of children and adolescents (*Q* = 7.670, *p* = 0.022). In contrast, there was no significant difference in the prevalence of sleep disturbances as assessed by different scales on sleep disturbances (*Q* = 10.281, *p* = 0.068). Meta-regression analyses revealed that mean age (*B* = −0.20, *z* = −53.79, *p* < 0.001), study quality assessment (*B* = −19, *z* = −34.38, *p* < 0.001) and percentage of men (*B* = −0.37, *z* = −16.79, *p* < 0.001) showed negative associations with the prevalence of sleep disturbances (Supplementary Figs. 2–4). In contrast, the time of the survey (*B* = 1.82, *z* = 34.02, *p* < 0.001) showed a positive association with the prevalence of sleep disturbances (Supplementary Fig. 5).

**Table 2:** Subgroup analysis of the prevalence of sleep disturbances.

Subgroup	Categories (no. of studies)	Event	Total	Prevalence (%)	95% CI (%)	*I*^2^ (%)	*p* value within subgroup	*Q* (*p* values across subgroups)
*Type of data collection*								**7.949 (*p*** = **0.005)**
Self-report	30	27,609	140,214	24.4	18.7–31.0	99.800	<0.001	
Parent-report	27	19,423	66,387	40.2	31.4–49.7	99.733	<0.001	
*Region*								**23.656 (*p*** < **0.001)**
America	5	1339	4830	40.9	16.5–70.8	99.306	<0.001	
Asia	29	11,306	72,422	21.8	16.5–28.2	99.802	<0.001	
Asia and Europe	4	805	1797	59.2	22.7–87.8	98.953	<0.001	
Europe	12	13,054	33,178	40.1	27.8–53.7	99.783	<0.001	
Oceania	3	1793	6540	27.5	16.7–41.9	99.213	<0.001	
South America	4	3992	10,647	50.7	39.0–62.4	97.528	<0.001	
*Children/adolescents*								**7.670 (*p*** = **0.022)**
Adolescents	20	9688	80,249	21.1	13.4–31.6	99.802	<0.001	
Both	19	24,803	78,898	33.2	26.3–40.9	99.793	<0.001	
Children	18	12,514	47,454	42.2	31.7–53.4	99.693	<0.001	
*Scale on sleep disturbances*								10.281 (*p* = 0.068)
CSHQ	6	2451	9160	59.2	28.9–83.8	99.770	<0.001	
ISI	5	2417	10,360	23.5	12.4–40.0	99.576	<0.001	
PSQI	5	5736	24,386	33.6	20.8–49.3	99.744	<0.001	
Single question	21	22,527	110,024	24.2	17.0–33.3	99.846	<0.001	
SDSC	9	6403	14,691	50.4	30.0–70.6	99.735	<0.001	
YSIS	2	842	3149	22.5	6.3–55.4	99.578	<0.001	

Bolded value, <0.05.

*SDSC* sleep disturbance scale for children, *PSQI* Pittsburgh sleep quality index, *CSHQ* children’s sleep habit, *ISI* insomnia severity. index.

## Discussion

This systematic review and meta-analysis found that the worldwide prevalence of sleep disturbances in children and adolescents was 34% (95% CI: 28–41%) during the COVID-19 pandemic, which is lower than the corresponding figures in two previous meta-analyses which reported 54% (95% CI: 50–57%) [15] and 44% (95% CI: 21%, 68%) [8], respectively. However, it should be noted that in the first meta-analysis [15] children and adolescents with neurobehavioral disorders were included, which could increase the prevalence of sleep disturbances; further, only 5 studies were included which could only provide preliminary findings. In another meta-analysis [8], the prevalence of sleep disorders, rather than sleep disturbances more broadly, was examined. Hence, direct comparisons between studies must be made with caution. In addition, our finding was higher than the corresponding figure (26%; 95% CI: 24–27%) in adolescents [26] and the general population (15%; 95% CI: 12.1–18.5%) [27] in China prior to the COVID-19 pandemic. Possible reasons for increased sleep disturbances in children and adolescents included fear caused by the pandemic and related preventive measures such as closures of school and recreation facilities, social distancing from their peers, and compulsory home quarantine, all of which could increase the risk of mental health problems including sleep disturbances in this population [8, 15].

In this meta-analysis, parent-reported sleep disturbance prevalence was higher than self-reported sleep disturbance by children and adolescents. This is consistent with previous findings [28, 29]. Other research [30] has found that when assessing health/disease status, external raters (e.g. physicians and caregivers) are more likely to focus on patients’ objective symptoms and disease diagnoses, whereas patients tend to focus on their subjective symptoms, functional limitations, and quality of life; consequently, discordances between self-report and external assessments are likely. Hence, sleep disturbances in children and adolescents such as difficulties falling asleep during the pandemic and home isolation were more likely to be identified by their parents. Besides, based on the duration of online activities among adolescents and children during the pandemic [31, 32], parents could also ascertain the sleep disturbances in their children objectively.

Younger children were associated with a higher risk of sleep disturbances, which was the opposite result compared to the findings reported prior to the pandemic [26]. Several factors might account for this finding. Younger children usually spend more time doing outdoor and recreational activities and less time studying than older children and adolescents. During the pandemic, however, young children were relatively more deprived of outdoor activities and social groups compared with adolescents who had spent more time in school [15]. Additionally, children were often more fearful due to their lack of understanding of the COVID-19 pandemic’s effects. All these factors might increase the risk of having mental health problems including sleep disturbances [33].

The prevalence of sleep disturbance was significantly higher in studies jointly conducted across Asia and Europe compared to those conducted in Asia, Europe, America, Oceania, and South America alone. There is an substantial inequalities in health care among children and adolescents across countries due to different country-specific socioeconomic and demographic indicators [34], the severity of the pandemic, and access to child mental health services [35], all of which could result in variations in prevalence of sleep disturbances across the regions.

The prevalence of sleep disturbances has increased in more recent surveys, which is consistent with the findings of prospective studies on insomnia symptoms among college students in China [36] and adults living in the United States [37]. Most of the included studies were conducted in 2020 and the first half of the year 2021, during the first peak of the pandemic wave. The prevalence of insomnia symptoms increased dramatically during the initial months of the pandemic [38]. Most children and adolescents experienced stricter public health measures over this period, such as the change from classroom teaching to online classes and reduced outdoor activities, which could substantially affect their sleep/wake schedules and increase their risk of insomnia symptoms [39].

The meta-analysis also found that girls were more likely to have sleep disturbances than boys, which is consistent with the notion that girls usually have a higher risk of mental health-related problems [11, 40]. Possible reasons for the gender difference may include an increased risk of interpersonal stressors, more experience of violence, increased screen time, and reduced outdoor activities during the COVID-19 pandemic in girls [33, 41].

We found a negative relationship between study quality and sleep disturbance prevalence. High-quality studies are usually associated with random sampling, well-trained interviewers, and well-validated measures, all of which could reduce the likelihood of false detection of sleep disturbances and as such, result in a relatively lower prevalence compared to poor-quality studies. In this meta-analysis, most of the included studies were rated as moderate study quality, and therefore high-quality studies that use random sampling, well-trained interviewers, and well-validated measures should be conducted in the future.

The strengths of this meta-analysis include the large number of studies and pooled sample size from both international and Chinese databases. Considering that COVID-19 was first reported in China and that many of the relevant studies were published in Chinese-language journals, it is important to include studies from Chinese-language databases. Several limitations should be noted. First, most of the studies conducted in the early stage of the pandemic (February–March 2020) were conducted in East Asia. Second, most studies were cross-sectional in nature; therefore, the dynamic changes in sleep disturbance prevalence between different periods could not be examined. Third, convenience sampling was used in most studies, which limits the representativeness of the study sample. Fourth, factors associated with sleep disturbances, such as academic pressure at school and psychiatric comorbidities, were not examined due to insufficient data. Finally, heterogeneity is unavoidable when conducting the meta-analysis of epidemiological studies [42, 43], even if subgroup meta-regression analyses were performed.

In conclusion, sleep disturbances were common in children and adolescents during the COVID-19 pandemic, particularly in children. Considering the negative impact of sleep disturbances on daily life, academic performance, and well-being, appropriate prevention and treatment measures should be implemented for this vulnerable population.

### Supplementary information

supplementary materials

## Data Availability

The data of the investigation will be made publicly available if necessary.
