# Supplementary material for: Prevalence of sleep disturbances in children and adolescents during COVID-19 pandemic: a meta-analysis and systematic review of epidemiological surveys
Source: Transl Psychiatry. 2024 Jan 8;14:12. doi: 10.1038/s41398-023-02654-5 (PMC10774396; doi:10.1038/s41398-023-02654-5)
Supplement: Supplementary file 1 — supplementary materials [file 41398_2023_2654_MOESM1_ESM.docx]

**Supplementary materials**

**Figure legends**

Figure 1. Publication bias of included studies reporting prevalence of insomnia. (Plotted by random effects)

Figure 2. Meta-regression of male percentage

Figure 3. Meta-regression of mean age

Figure 4. Meta-regression of quality evaluation score

Figure 5. Meta-regression of time of survey

Supplementary Table 1 search methodology of databases

| PubMed | (Sleep Initiation and Maintenance Disorders [MeSH Terms] OR sleep disturbance OR insomnia OR sleep problem OR sleep disorder OR sleep symptom OR sleep*)  AND (adolescent [MeSH Terms] OR child OR children OR preschool OR pediatrics OR infants OR toddlers)  AND (2019-ncov* OR 2019ncov* OR 2019n-cov* OR coronaviru* OR corona viru* OR covid OR covid-19 OR covid19* OR ncov* OR novel cov* OR ncov* OR covid-2019 OR covid2019 OR SARS-COV-2* OR SARS-COV2* OR SARS COV-2* OR SARS COV2* OR SARS COV19 OR SARS COV-19 OR SARS COV-19 OR SARS-COV-2019 OR SARS COV 2019 OR SARS COV-2019 OR severe acute respiratory syndrome or severe acute respiratory disease)  AND (epidemiology OR prevalence OR rate) |
| --- | --- |
| Web of science | (Adolescent* OR Teen* OR Teenager* OR Youth* OR child OR children OR preschool OR pediatrics OR infants OR toddlers)  (Disorders of Initiating and Maintaining Sleep OR Early Awakening OR Sleep Initiation Dysfunction OR Sleeplessness OR Insomnia OR sleep disturbance OR sleep problem OR sleep disorder OR sleep symptom OR sleep*)  (2019-ncov* OR 2019ncov* OR 2019n-cov* OR coronaviru* OR corona viru* OR covid OR covid-19 OR covid19* OR ncov* OR novel cov* OR covid-2019 OR covid2019 OR SARS-COV-2* OR SARS-COV2* OR SARS COV-2* OR SARS COV2* OR SARS COV19 OR SARS COV-19 OR SARS-COV-2019 OR SARS COV 2019 OR SARS COV-2019 OR severe acute respiratory syndrome or severe acute respiratory disease) |
| PsycINFO | (Adolescent* OR Teen* OR Teenager* OR Youth* OR child OR children OR preschool OR pediatrics OR infants OR toddlers)  (Disorders of Initiating and Maintaining Sleep OR Early Awakening OR Sleep Initiation Dysfunction OR Sleeplessness OR Insomnia OR sleep disturbance OR sleep problem OR sleep disorder OR sleep symptom OR sleep*)  (2019-ncov* OR 2019ncov* OR 2019n-cov* OR coronaviru* OR corona viru* OR covid OR covid-19 OR covid19* OR ncov* OR novel cov* OR covid-2019 OR covid2019 OR SARS-COV-2* OR SARS-COV2* OR SARS COV-2* OR SARS COV2* OR SARS COV19 OR SARS COV-19 OR SARS-COV-2019 OR SARS COV 2019 OR SARS COV-2019 OR severe acute respiratory syndrome or severe acute respiratory disease) |
| CNKI | (“睡眠习惯” or “睡眠紊乱”or “失眠” or “睡眠” or “睡眠障碍”) and (“率” or”患病情况”or “调查” or ”流行病学”) and (“儿童” or ”青少年” or “儿科” or ”婴儿” or “幼儿”) and (“新冠肺炎” or “COVID-19”) |
| WANFANG | (“睡眠习惯” or “睡眠紊乱”or “失眠” or “睡眠” or “睡眠障碍”) and (“率” or”患病情况”or “调查” or ”流行病学”) and (“儿童” or ”青少年” or “儿科” or ”婴儿” or “幼儿”) and (“新冠肺炎” or “COVID-19”) |

Supplementary Table 2. Quality assessment of included studies

|  |  | **1. Clearly defined target population** | **2. Probability sampling or entire population** | **3. Response rate equal to or greater than 80%** | **4. clearly described non-responders** | **5. The sample representative of the target population** | **6. Standardized data collection methods** | **7. Validated criteria used to measure sleep disturbance** | **8. Prevalence estimates with confidence intervals and detailed by subgroups (if applicable)** | **Total score** |
| --- | --- | --- | --- | --- | --- | --- | --- | --- | --- | --- |
| 1 | Liang et al 2020 | 1 | 1 | 0 | 0 | 1 | 1 | 1 | 0 | 5 |
| 2 | Wang et al 2020 | 1 | 0 | 0 | 0 | 1 | 1 | 1 | 0 | 4 |
| 3 | Yang et al 2020 | 1 | 0 | 1 | 0 | 1 | 1 | 1 | 0 | 5 |
| 4 | Bruni et al 2021 | 1 | 0 | 0 | 0 | 1 | 1 | 1 | 0 | 4 |
| 5 | Chi et al 2021 | 1 | 1 | 1 | 1 | 1 | 1 | 1 | 0 | 7 |
| 6 | Dondi et al 2020 | 1 | 0 | 0 | 1 | 1 | 1 | 1 | 0 | 5 |
| 7 | WearickSilva et al 2021 | 1 | 0 | 0 | 0 | 1 | 1 | 1 | 0 | 4 |
| 8 | Eyuboglu et al 2021 | 1 | 1 | 0 | 0 | 1 | 1 | 1 | 0 | 5 |
| 9 | Fidanci et al 2021 | 1 | 0 | 0 | 0 | 1 | 1 | 1 | 0 | 4 |
| 10 | LopezGil et al 2021 | 1 | 1 | 0 | 0 | 1 | 1 | 1 | 1 | 6 |
| 11 | Hu et al 2021 | 1 | 1 | 0 | 0 | 1 | 1 | 1 | 0 | 5 |
| 12 | Li et al 2021 | 1 | 0 | 0 | 0 | 1 | 1 | 1 | 0 | 4 |
| 13 | Liu Y. et al 2021 | 1 | 1 | 1 | 0 | 1 | 1 | 1 | 0 | 6 |
| 14 | Liu, Z et al 2021 | 1 | 1 | 0 | 0 | 1 | 1 | 1 | 0 | 5 |
| 15 | Moulin et al 2021 | 1 | 0 | 0 | 0 | 1 | 1 | 1 | 0 | 4 |
| 16 | Nakachi et al 2021 | 1 | 1 | 1 | 1 | 1 | 1 | 1 | 0 | 7 |
| 17 | Osmanov et al 2021 | 1 | 0 | 0 | 1 | 1 | 1 | 1 | 0 | 5 |
| 18 | Weingart et al 2021 | 1 | 1 | 0 | 0 | 1 | 1 | 1 | 0 | 5 |
| 19 | Resendiz-Aparicio et al 2021 | 1 | 0 | 0 | 0 | 1 | 1 | 1 | 0 | 4 |
| 20 | Ventura et al 2021 | 1 | 1 | 0 | 0 | 1 | 1 | 1 | 0 | 5 |
| 21 | Wang, L et al 2021 | 1 | 1 | 0 | 0 | 1 | 1 | 1 | 0 | 5 |
| 22 | Zhai et al 2021 | 1 | 1 | 0 | 0 | 1 | 1 | 1 | 0 | 5 |
| 23 | Zhou et al 2020 | 1 | 0 | 1 | 0 | 1 | 1 | 1 | 0 | 5 |
| 24 | Li et al 2021 | 1 | 1 | 0 | 0 | 1 | 1 | 1 | 0 | 5 |
| 25 | Luca et al 2021 | 1 | 0 | 0 | 0 | 1 | 1 | 1 | 0 | 4 |
| 26 | Kumar et al 2020 | 1 | 0 | 0 | 0 | 1 | 1 | 1 | 0 | 4 |
| 27 | Szwarcwald et al 2021 | 1 | 1 | 0 | 0 | 1 | 1 | 1 | 0 | 5 |
| 28 | Ding et al 2022 | 1 | 1 | 1 | 1 | 1 | 1 | 1 | 0 | 7 |
| 29 | Li et al 2022 | 1 | 0 | 0 | 0 | 1 | 1 | 1 | 0 | 4 |
| 30 | Wang et al 2022 | 1 | 1 | 1 | 0 | 1 | 1 | 1 | 0 | 6 |
| 31 | Xu et al 2022 | 1 | 1 | 0 | 0 | 1 | 1 | 1 | 0 | 5 |
| 32 | Zhao et al 2022 | 1 | 1 | 1 | 0 | 1 | 1 | 1 | 0 | 6 |
| 33 | Bacaro et al 2021 | 1 | 0 | 0 | 0 | 1 | 1 | 1 | 0 | 4 |
| 34 | Bacaro et al 2022 (Bacaro et al., 2022) | 1 | 0 | 0 | 0 | 1 | 1 | 1 | 0 | 4 |
| 35 | Becker et al 2021 | 1 | 0 | 0 | 0 | 1 | 1 | 1 | 0 | 4 |
| 36 | Bothe et al 2022(Bothe et al., 2022) | 1 | 0 | 0 | 0 | 1 | 1 | 1 | 0 | 4 |
| 37 | Gendler et al 2022 | 1 | 0 | 0 | 0 | 1 | 1 | 1 | 0 | 4 |
| 38 | Ho et al 2022 | 1 | 0 | 0 | 0 | 1 | 1 | 1 | 0 | 4 |
| 49 | Kaltschik et al 2022 | 1 | 0 | 0 | 0 | 1 | 1 | 1 | 0 | 4 |
| 40 | Lima et al 2022 | 1 | 0 | 0 | 0 | 1 | 1 | 1 | 0 | 4 |
| 41 | Ma et al 2021 | 1 | 0 | 0 | 0 | 1 | 1 | 1 | 0 | 4 |
| 42 | Mackenzie et al 2021 | 1 | 0 | 0 | 0 | 1 | 1 | 1 | 0 | 4 |
| 43 | Mensi et al 2022 | 1 | 0 | 1 | 0 | 1 | 1 | 1 | 0 | 5 |
| 44 | Moitra et al 2022 | 1 | 1 | 1 | 0 | 1 | 1 | 1 | 0 | 6 |
| 45 | Monnier et al 2021 | 1 | 0 | 0 | 0 | 1 | 1 | 1 | 0 | 4 |
| 46 | Pieh et al 2022 | 1 | 0 | 0 | 0 | 1 | 1 | 1 | 0 | 4 |
| 47 | Sánchez-Ferrer et al 2022 | 1 | 0 | 0 | 0 | 1 | 1 | 1 | 0 | 4 |
| 48 | Scarselli et al 2022 | 1 | 0 | 0 | 0 | 0 | 1 | 1 | 0 | 3 |
| 59 | Sen et al 2021 | 1 | 0 | 0 | 0 | 1 | 1 | 1 | 0 | 4 |
| 50 | Silver et al 2022 | 1 | 0 | 0 | 0 | 0 | 1 | 1 | 0 | 3 |
| 51 | (Ustuner Top and Cam, 2022) | 1 | 1 | 0 | 0 | 1 | 1 | 1 | 0 | 5 |
| 52 | Van et al 2022 | 1 | 1 | 0 | 0 | 1 | 1 | 1 | 0 | 5 |
| 53 | Xue et al 2022 | 1 | 1 | 0 | 0 | 1 | 1 | 1 | 0 | 5 |
| 54 | Zhan et al 2022 | 1 | 1 | 0 | 0 | 1 | 1 | 1 | 0 | 5 |
| 55 | Zhang et al 2021 | 1 | 1 | 0 | 0 | 1 | 1 | 1 | 0 | 5 |
| 56 | Zhou et al 2021 | 1 | 0 | 1 | 1 | 1 | 1 | 1 | 0 | 6 |
| 57 | Zhu et al 2022 | 1 | 1 | 1 | 1 | 1 | 1 | 1 | 0 | 7 |

Figure 1. Publication bias of included studies reporting prevalence of insomnia. (Plotted by random effects)


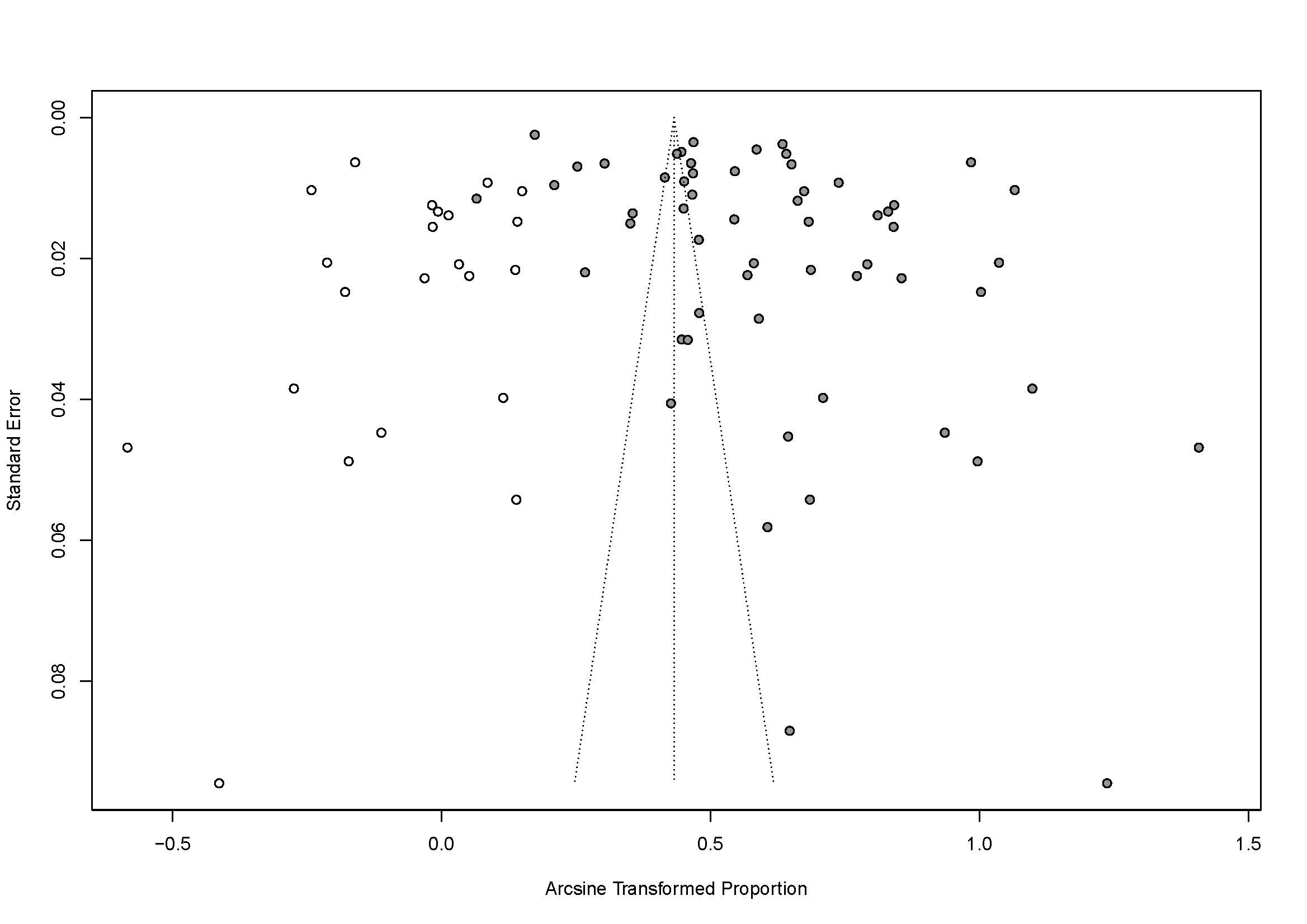


Figure 2. Meta-regression of male percentage


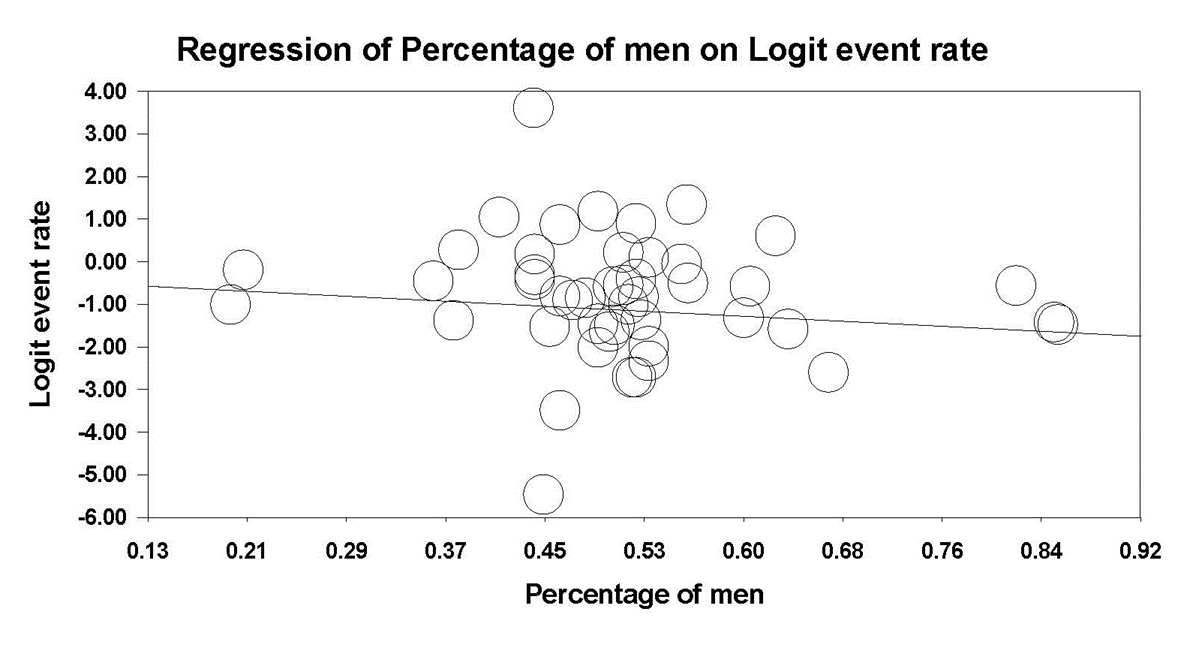


Figure 3. Meta-regression of mean age


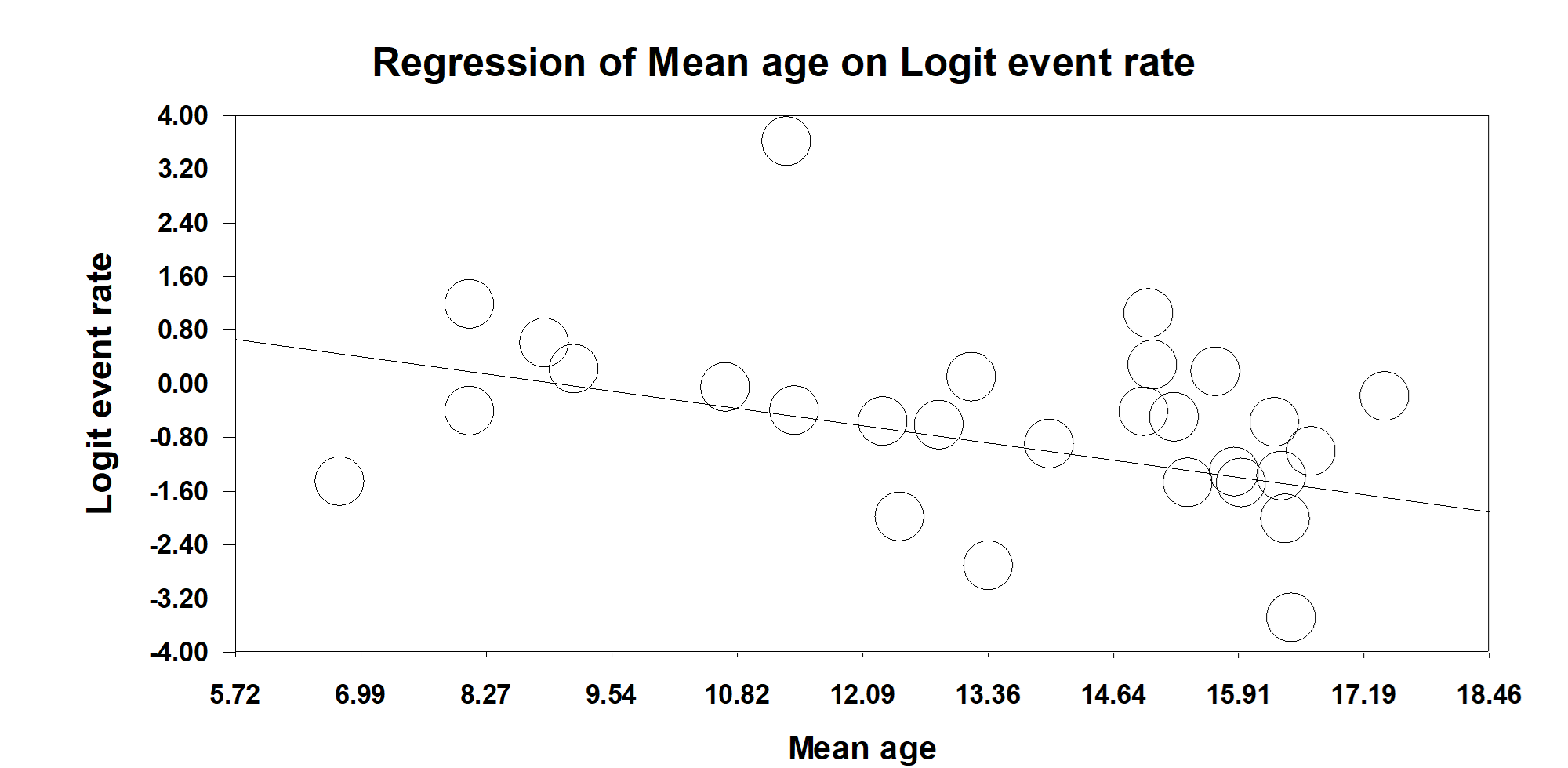


Figure 4. Meta-regression of quality evaluation score


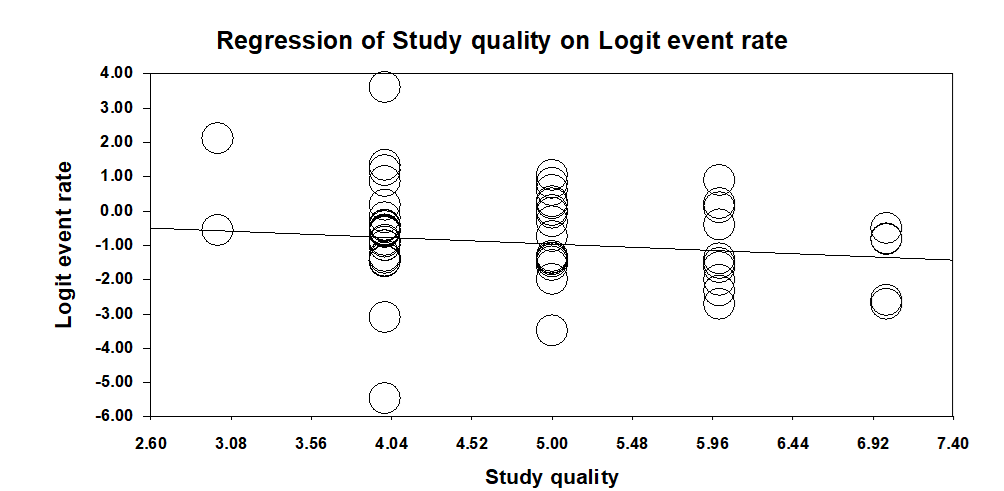


Figure 5. Meta-regression of time of survey


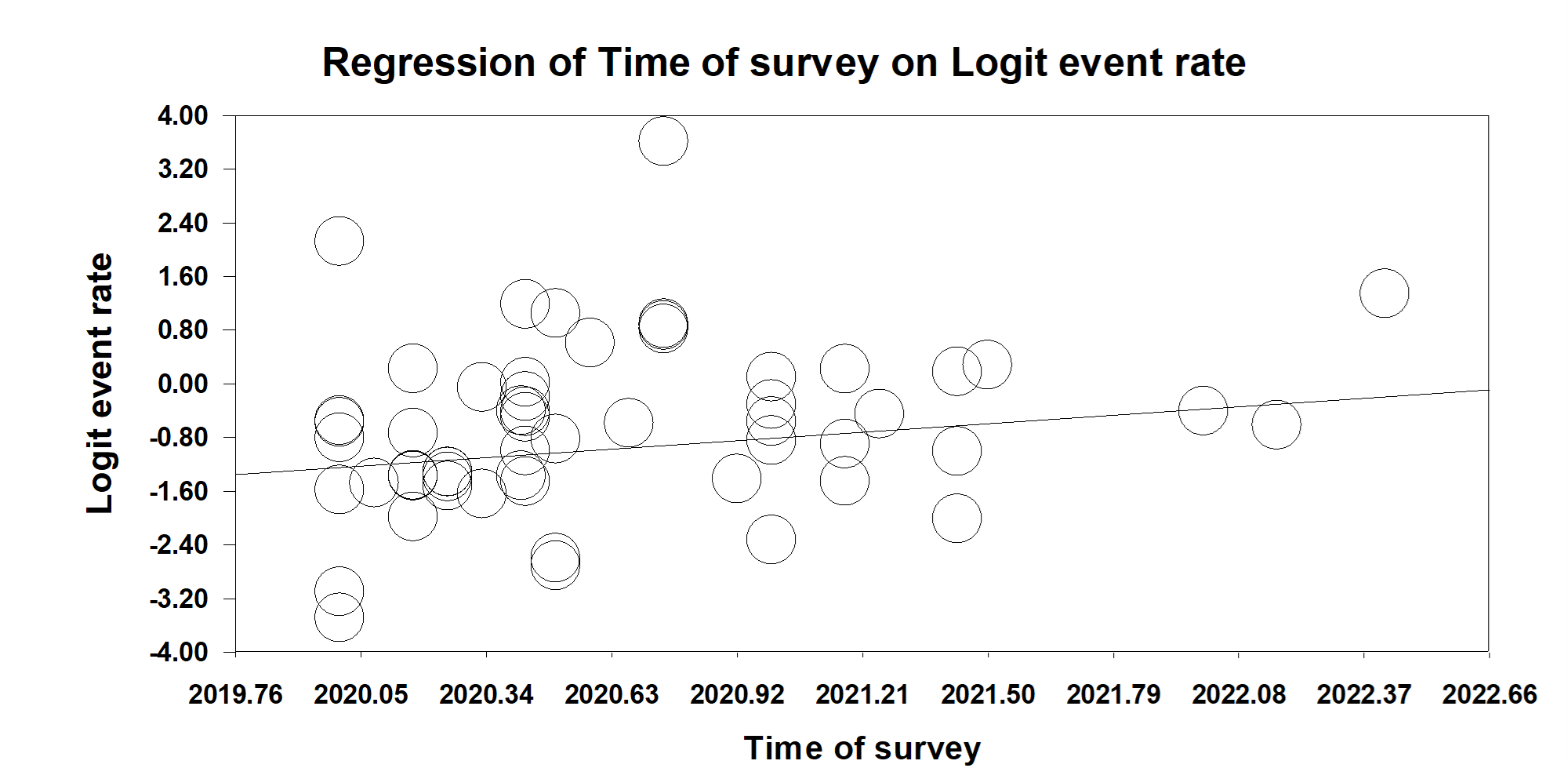


2020 PRISMA checklist

| **Section and Topic** | **Item #** | **Checklist item** | **Location where item is reported** |
| --- | --- | --- | --- |
| **TITLE** | | |  |
| Title | 1 | Identify the report as a systematic review. | 1 |
| **ABSTRACT** | | |  |
| Abstract | 2 | See the PRISMA 2020 for Abstracts checklist. | 4 |
| **INTRODUCTION** | | |  |
| Rationale | 3 | Describe the rationale for the review in the context of existing knowledge. | 5 |
| Objectives | 4 | Provide an explicit statement of the objective(s) or question(s) the review addresses. | 6 |
| **METHODS** | | |  |
| Eligibility criteria | 5 | Specify the inclusion and exclusion criteria for the review and how studies were grouped for the syntheses. | 6 |
| Information sources | 6 | Specify all databases, registers, websites, organisations, reference lists and other sources searched or consulted to identify studies. Specify the date when each source was last searched or consulted. | 6 |
| Search strategy | 7 | Present the full search strategies for all databases, registers and websites, including any filters and limits used. | 6 |
| Selection process | 8 | Specify the methods used to decide whether a study met the inclusion criteria of the review, including how many reviewers screened each record and each report retrieved, whether they worked independently, and if applicable, details of automation tools used in the process. | 6-7 |
| Data collection process | 9 | Specify the methods used to collect data from reports, including how many reviewers collected data from each report, whether they worked independently, any processes for obtaining or confirming data from study investigators, and if applicable, details of automation tools used in the process. | 7 |
| Data items | 10a | List and define all outcomes for which data were sought. Specify whether all results that were compatible with each outcome domain in each study were sought (e.g. for all measures, time points, analyses), and if not, the methods used to decide which results to collect. | 8 |
|  | 10b | List and define all other variables for which data were sought (e.g. participant and intervention characteristics, funding sources). Describe any assumptions made about any missing or unclear information. | 8 |
| Study risk of bias assessment | 11 | Specify the methods used to assess risk of bias in the included studies, including details of the tool(s) used, how many reviewers assessed each study and whether they worked independently, and if applicable, details of automation tools used in the process. | 8 |
| Effect measures | 12 | Specify for each outcome the effect measure(s) (e.g. risk ratio, mean difference) used in the synthesis or presentation of results. | 8 |
| Synthesis methods | 13a | Describe the processes used to decide which studies were eligible for each synthesis (e.g. tabulating the study intervention characteristics and comparing against the planned groups for each synthesis (item #5)). | 8-9 |
|  | 13b | Describe any methods required to prepare the data for presentation or synthesis, such as handling of missing summary statistics, or data conversions. | 8-9 |
|  | 13c | Describe any methods used to tabulate or visually display results of individual studies and syntheses. | 8-9 |
|  | 13d | Describe any methods used to synthesize results and provide a rationale for the choice(s). If meta-analysis was performed, describe the model(s), method(s) to identify the presence and extent of statistical heterogeneity, and software package(s) used. | 8-9 |
|  | 13e | Describe any methods used to explore possible causes of heterogeneity among study results (e.g. subgroup analysis, meta-regression). | 8-9 |
|  | 13f | Describe any sensitivity analyses conducted to assess robustness of the synthesized results. | 8-9 |
| Reporting bias assessment | 14 | Describe any methods used to assess risk of bias due to missing results in a synthesis (arising from reporting biases). | 8-9 |
| Certainty assessment | 15 | Describe any methods used to assess certainty (or confidence) in the body of evidence for an outcome. | 8-9 |
| **RESULTS** | | |  |
| Study selection | 16a | Describe the results of the search and selection process, from the number of records identified in the search to the number of studies included in the review, ideally using a flow diagram. | 9 |
|  | 16b | Cite studies that might appear to meet the inclusion criteria, but which were excluded, and explain why they were excluded. | 9 |
| Study characteristics | 17 | Cite each included study and present its characteristics. | 22-23 |
| Risk of bias in studies | 18 | Present assessments of risk of bias for each included study. | 26-27 |
| Results of individual studies | 19 | For all outcomes, present, for each study: (a) summary statistics for each group (where appropriate) and (b) an effect estimate and its precision (e.g. confidence/credible interval), ideally using structured tables or plots. | 9 |
| Results of syntheses | 20a | For each synthesis, briefly summarise the characteristics and risk of bias among contributing studies. | 9 |
|  | 20b | Present results of all statistical syntheses conducted. If meta-analysis was done, present for each the summary estimate and its precision (e.g. confidence/credible interval) and measures of statistical heterogeneity. If comparing groups, describe the direction of the effect. | 9 |
|  | 20c | Present results of all investigations of possible causes of heterogeneity among study results. | 9-10 |
|  | 20d | Present results of all sensitivity analyses conducted to assess the robustness of the synthesized results. | 9-1- |
| Reporting biases | 21 | Present assessments of risk of bias due to missing results (arising from reporting biases) for each synthesis assessed. | 9-10 |
| Certainty of evidence | 22 | Present assessments of certainty (or confidence) in the body of evidence for each outcome assessed. | 9-10 |
| **DISCUSSION** | | |  |
| Discussion | 23a | Provide a general interpretation of the results in the context of other evidence. | 10 |
|  | 23b | Discuss any limitations of the evidence included in the review. | 12 |
|  | 23c | Discuss any limitations of the review processes used. | 12 |
|  | 23d | Discuss implications of the results for practice, policy, and future research. | 10-12 |
| **OTHER INFORMATION** | | |  |
| Registration and protocol | 24a | Provide registration information for the review, including register name and registration number, or state that the review was not registered. | 6 |
|  | 24b | Indicate where the review protocol can be accessed, or state that a protocol was not prepared. | 6 |
|  | 24c | Describe and explain any amendments to information provided at registration or in the protocol. | 6 |
| Support | 25 | Describe sources of financial or non-financial support for the review, and the role of the funders or sponsors in the review. | 1 |
| Competing interests | 26 | Declare any competing interests of review authors. | 2 |
| Availability of data, code and other materials | 27 | Report which of the following are publicly available and where they can be found: template data collection forms; data extracted from included studies; data used for all analyses; analytic code; any other materials used in the review. | 6-7 |
